# Conservative Kidney Management in Elderly Patients Not Receiving Dialysis: How to Proceed?

**DOI:** 10.34067/KID.0000000977

**Published:** 2025-10-30

**Authors:** Abdullah Al-Muhaiteeb

**Affiliations:** Nephrology Division, Al Amiri Hospital, Kuwait City, Kuwait

**Keywords:** dialysis, geriatric nephrology, hemodialysis, peritoneal dialysis, conservative management

The number of elderly patients developing ESKD has been steadily increasing.^1,2^ In 2022, the adjusted incidence rate was 1461 per million population among individuals aged 75 years and older.^3^ Despite advances in technology and the growing availability of dialysis options, conservative kidney management (CKM) remains an underutilized approach. Many of these older patients are frail and burdened with multiple comorbidities, raising important questions about the most appropriate and compassionate course of care. According to the Kidney Disease Improving Global Outcomes 2024 guidelines,^4^ conservative renal care is a reasonable option to consider for patients who do not wish to pursue KRT.

In this issue of *Kidney360*, Kobayashi *et al.*^5^ conducted a retrospective cohort study using data from a national health examination cohort, spanning from April 2014 to May 2023. The data, provided by DeCS HealthCare Inc. (Tokyo, Japan), included health insurance claims records. The study population consisted of individuals aged 75 years or older with an eGFR of <8 ml/min who did not receive KRT.

The authors divided patients into two groups: the CKM group and the non-CKM group. The CKM group included those who underwent no procedures related to KRT, while the non-CKM group consisted of patients who had undergone procedures such as vascular access surgery or peritoneal dialysis (PD) catheter insertion.

For statistical analysis, a Cox proportional hazards model was used to evaluate mortality, while age, sex, body mass index, frailty, and renal function were assessed as factors influencing CKM selection. In addition, a Poisson regression model was used to calculate incidence rate ratios for hospitalization. Frailty was assessed using the Electronic Frailty Index. The study found that the CKM group had significantly lower hospitalization rates compared with the non-CKM group, without any significant effect on overall survival.

Although the study addresses a clinically important and relevant issue, which is the benefit of KRT in a vulnerable elderly population, it has significant flaws and limitations that threaten its internal validity. Most notably, the study population was drawn from a health checkup database, which may not be representative of the broader population of older adults with stage 5 CKD. Individuals who undergo routine health checkups are often healthier or more health conscious than those who do not, introducing a potential selection bias. As a result, the benefits of CKM may be overestimated, since the study participants likely had fewer comorbidities and better overall health than the general elderly CKD population.

Another limitation of the study is its use of administrative claims data, which often lacks detailed clinical information. Important factors such as patient preferences, clinical presentations, and the presence of uremic symptoms or heart failure were not available. The absence of this information may have led to incorrect classification of patients into the CKM or non-CKM groups and could also compromise the internal validity of the study's findings. In addition, the study did not explore the nature of care received by patients in the CKM group. For example, whether they received supportive care or palliative care. Clarifying this distinction would offer greater insight into patient well-being and the management of symptoms.

The study adjusted for several covariates, including age, sex, body mass index, and frailty. Nevertheless, unmeasured confounders may still be present. Factors such as socioeconomic status, access to health care, and patient preferences could influence both the choice of CKM and the outcomes but were not included in the analysis. Frailty was assessed using the Electronic Frailty Index based on administrative data, which may not fully capture the complexity of frailty as effectively as a comprehensive clinical assessment.

Unfortunately, CKM is sometimes seen as a passive approach or as simply “doing nothing.” In reality, it is an active, structured strategy that includes thorough symptom management, BP and fluid control, anemia treatment, dietary guidance, and psychosocial support, all without initiating dialysis.^6,7^

The study by Kobayashi *et al.* did not examine this type of care.^5^ Instead, it focused on outcomes such as mortality and hospitalization. Although these are important in this vulnerable population, other clinically meaningful outcomes must also be considered. These include quality of life, cognitive function, progression of frailty, psychologic well-being (such as depression), and levels of independence.

In addition, this study compared CKM with KRT. As nephrologists, we recognize that KRT encompasses several modalities, including conventional hemodialysis, home hemodialysis, and PD. Comparing CKM to a heterogeneous group of dialysis options is suboptimal and may introduce bias or lead to misinterpretation of outcomes. It is well established that home-based therapies, such as home hemodialysis and PD, often provide better outcomes for patients, including improved quality of life, greater independence, and lower hospitalization rates^8,9^

CKM can be an excellent option; when it is delivered within a shared decision-making framework, it can provide patients and their families with informed, realistic choices helping them avoid the physical, emotional, and financial burdens of dialysis when its expected benefits are minimal.

Although this study provides an interesting insight into CKM in the elderly population, it does not produce clinically meaningful results that would significantly influence current practice. Given the limitations outlined, the findings should be interpreted with appropriate caution. Future research should aim to be more methodologically robust and focus on outcomes that are clinically relevant to older adults, as previously noted. Further research is needed to better guide clinical decisions and provide additional guidance to health care providers and caregivers.

## Supplementary Material

## References

[1] Paraskevas KI Bessias N Koupidis SA Tziviskou E Mikhailidis DP Oreopoulos DG. Incidence of end-stage renal disease in the elderly: a steadily rising global socioeconomic epidemic. Int Urol Nephrol. 2010;42(2):523–525. doi:10.1007/s11255-009-9691-1 20020205

[2] Xie K Cao H Ling S, . Global, regional, and national burden of chronic kidney disease, 1990-2021: a systematic analysis for the global burden of disease study 2021. Front Endocrinol (Lausanne). 2025;16:1526482. doi:10.3389/fendo.2025.1526482 40110544 PMC11919670

[3] United States Renal Data System. USRDS 2024 Annual Data Report: Epidemiology of Kidney Disease in the United States; 2024.

[4] Kidney Disease Improving Global Outcomes (KDIGO) CKD Work Group. KDIGO 2024 clinical practice guideline for the evaluation and management of chronic kidney disease. Kidney Int. 2024;105(4S):S117–S314. doi:10.1016/j.kint.2023.10.018 38490803

[5] Kobayashi A Hirano K Okuda T, . Comprehensive conservative kidney management among older population: a nationwide administrative claims database analysis. Kidney360. 2025;6(10):1713–1721. doi:10.34067/KID.0000000923 40694417 PMC12778016

[6] O'Connor NR Kumar P. Conservative management of end-stage renal disease without dialysis: a systematic review. J Palliat Med. 2012;15(2):228–235. doi:10.1089/jpm.2011.0207 22313460 PMC3318255

[7] Brown MA Collett GK Josland EA Foote C Li Q Brennan FP. CKD in elderly patients managed without dialysis: survival, symptoms, and quality of life. Clin J Am Soc Nephrol. 2015;10(2):260–268. doi:10.2215/CJN.03330414 25614492 PMC4317735

[8] Chuasuwan A Pooripussarakul S Thakkinstian A Ingsathit A Pattanaprateep O. Comparisons of quality of life between patients underwent peritoneal dialysis and hemodialysis: a systematic review and meta-analysis. Health Qual Life Outcomes. 2020;18(1):191. doi:10.1186/s12955-020-01449-2 32552800 PMC7302145

[9] Ok E Demirci C Asci G, . Patient survival with extended home hemodialysis compared to In-Center conventional hemodialysis. Kidney Int Rep. 2023;8(12):2603–2615. doi:10.1016/j.ekir.2023.09.007 38106580 PMC10719649

