# Supplementary material for: Conservative Kidney Management in Elderly Patients Not Receiving Dialysis: How to Proceed?
Source: Kidney360. 2025 Oct 30;6(10):1645–6. doi: 10.34067/KID.0000000977 (PMC12778008; doi:10.34067/KID.0000000977)
Supplement: Supplementary file 1 [file kidney360-6-1645-s001.pdf]

ASN American Society of Nephrology
JASN Journal of the American Society of Nephrology
KIDNEY360 Accessing Our World From Every Angle
CJASN Clinical Journal of the American Society of Nephrology

# ASN Journal Disclosure Form

As per ASN journal policy, I have disclosed any financial relationships or commitments I have held in the past 36 months as included below. I have listed my Current Employer below to indicate there is a relationship requiring disclosure. If no relationship exists, my Current Employer is not listed.

A. Almuhaiteeb reports the following:
Employer: Ministry of Health Kuwait; Speakers Bureau: Amgen; Astrazeneca; GSK global; and Other Interests or Relationships: Dr. Al-Muhaiteeb completed the Baxter UHN EXPLORE Home Dialysis Fellowship 2015-2016.

I understand that the information above will be published within the journal article, if accepted, and that failure to comply and/or to accurately and completely report the potential financial conflicts of interest could lead to the following: 1) Prior to publication, article rejection, or 2) Post-publication, sanctions ranging from, but not limited to, issuing a correction, reporting the inaccurate information to the authors' institution, banning authors from submitting work to ASN journals for varying lengths of time, and/or retraction of the published work.

Name: Abdullah Almuhaiteeb
Manuscript ID: K360-2025-000840
Manuscript Title: Conservative kidney management in elderly patients not receiving dialysis: How to proceed ?
Date of Completion: August 15, 2025
Disclosure Updated Date: August 15, 2025
